# Prolonged bedrest reduces plasma high-density lipoprotein levels linked to markedly suppressed cholesterol efflux capacity

**DOI:** 10.1038/s41598-020-71921-y

**Published:** 2020-09-14

**Authors:** Athina Trakaki, Hubert Scharnagl, Markus Trieb, Michael Holzer, Helmut Hinghofer-Szalkay, Nandu Goswami, Gunther Marsche

**Affiliations:** 1 Division of Pharmacology, Otto Loewi Research Center for Vascular Biology, Immunology and Inflammation, Medical University of Graz, Universitätsplatz 4, 8010 Graz, Austria; 2 Institute of Medical and Chemical Laboratory Diagnostics, Medical University of Graz, Auenbruggerplatz 15, 8036 Graz, Austria; 3 BioTechMed Graz, Mozartgasse 12/II, 8010 Graz, Austria; 4 Division of Physiology, Otto Loewi Research Center for Vascular Biology, Immunology and Inflammation, Medical University of Graz, Neue Stiftingtalstrasse 6/D-5, 8010 Graz, Austria

**Keywords:** Enzymes, Lipids, Proteins, Biochemistry, Risk factors

## Abstract

Recent observations strongly connect high-density lipoproteins (HDL) function and levels with coronary heart disease outcomes and risk for infections and sepsis. To date, our knowledge of factors determining this connection is still very limited. The immobility associated with prolonged bedrest is detrimental to health, affecting several systems, including the cardiovascular, pulmonary, gastrointestinal, musculoskeletal and urinary. Effects of prolonged bedrest on the composition and functional properties of HDL remain elusive. We evaluated metrics of HDL composition and function in healthy male volunteers participating in a randomized, crossover head-down bedrest study. We observed that HDL cholesterol efflux capacity was profoundly decreased during bedrest, mediated by a bedrest associated reduction in plasma levels of HDL-cholesterol and major apolipoproteins (apo) apoA-I and apoA-II. Paraoxonase activity, plasma anti-oxidative capacity and the activities of lecithin-cholesterol acyltransferase and cholesteryl ester transfer protein were not affected. No change was observed in the content of HDL-associated serum amyloid A, a sensitive marker of inflammation. Resistive vibration exercise countermeasure during bedrest did not correct impaired cholesterol efflux capacity and only tended to increase arylesterase activity of HDL-associated paraoxonase. In conclusion, prolonged bedrest reduces plasma HDL levels linked to markedly suppressed HDL cholesterol efflux capacity. Resistive vibration exercise during bedrest did not correct HDL levels and impaired cholesterol efflux capacity.

## Introduction

Physical inactivity or bedrest during hospitalization has been proposed as a primary factor contributing to the functional decline of hospitalized patients^1^. In healthy older adults, as little as 10 days of bedrest is associated with considerable loss of strength and aerobic capacity, as well as with a tendency towards reduced physical activity that can last weeks^1^. Prolonged bedrest leads to increased heart rate and changes in body vascular resistance^2^ and increases the risk of hospital-acquired pneumonia, an important cause of morbidity and mortality in hospitalized patients, which is often caused by bacterial infection^3, 4^.

The importance of high-density lipoproteins (HDL) function in cardiovascular disease has become a topic of major interest over the last few years. HDL are thought to be cardioprotective, attributable to various functions including promoting the removal of excess cholesterol from plaque macrophage foam cells, which may lead to cholesterol reduction in the atherosclerotic plaque^5–7^. Recent observations strongly connect HDL function with coronary heart disease outcomes and contribute to the understanding of factors that change HDL functionality^5, 6^. From an evolutionary point of view, lipoproteins are not only described as lipid transporters but also display important functions in many aspects of immunity. Of all lipoproteins, HDL have the highest affinity for binding and neutralizing pathogen-associated lipids (e.g. lipopolysaccharide and lipoteichoic acid)^8, 9^, which mediate excessive immune activation in bacterial infections^9–11^; a fact that could also be of particular interest in septic conditions^12^. Specifically, an inverse association of HDL-cholesterol with morbidity, sepsis severity^9^ and death from infection or other non-cardiovascular causes^13^ is observed, while infusion of reconstituted HDL or HDL mimetic particles decrease morbidity and mortality in experimental sepsis models^14^. HDL have also been reported to have anti-parasite action^9^. Although literature on HDL and viral infections is debating^15^, HDL have been reported to prevent virus penetration, accounting for a modest, yet significant portion of the anti-viral activity of serum^16^. Specifically, apolipoprotein (apo) A-I, the major protein component of HDL, has been reported to exert potent anti-viral effects upon herpes simplex virus infection^17^ and to inhibit cell fusion in human immunodeficiency virus type 1 infected cells^18^. Previous studies provided evidence that prolonged bedrest affects lipoprotein metabolism^19, 20^, but its effect on the quantity and quality of HDL particles remains elusive. Head-down tilt bedrest studies comprise an important, ground-based spaceflight analogue, to understand physiological changes that occur during long-duration space flights or in bedridden individuals^21^.

Here we assessed whether prolonged bedrest affects HDL-related biomarkers, including HDL-cholesterol levels, cholesterol efflux capacity, paraoxonase activity, anti-oxidative capacity and the activities of lecithin-cholesterol acyltransferase (LCAT) and cholesteryl ester transfer protein (CETP) in subjects participating in the prospective, randomized, crossover ‘Medium duration Nutrition and vibration eXercise’ study^21^.

## Results

### Effects of prolonged bedrest on HDL metabolism and function

**Figure 1:**
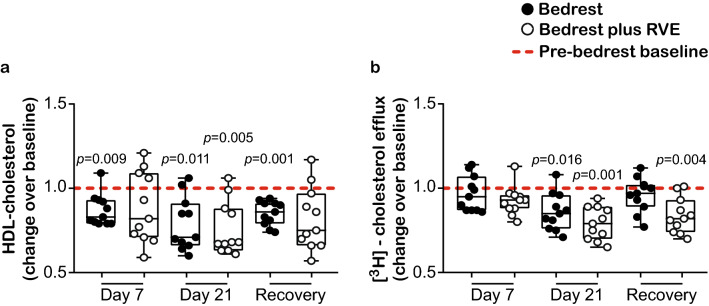
Effects of prolonged bedrest on HDL metabolism and function. Different metrics of HDL metabolism and function were evaluated for the bedrest (n = 11) and the bedrest plus RVE (n = 11) groups. (**a**) Plasma HDL-cholesterol levels were evaluated by a commercially available kit. (**b**) The ability of HDL to promote [^3^H]-cholesterol efflux from macrophages was evaluated. [^3^H]-cholesterol-labeled J774.2 cells were incubated with apoB-depleted plasma (2.8%) for 3 h and the effluxed [^3^H]-cholesterol was quantified. Cholesterol efflux is expressed as radioactivity in the cell culture supernatant relative to total radioactivity (in the cell culture supernatant and cells) of two independent experiments, measured in duplicates. (**a**,**b**) Differences between pre-bedrest baseline and day 7 of bedrest, day 21 of bedrest and one day recovery, as well as differences between the two groups at each time point, were analyzed with RM one-way ANOVA using the Sidak’s multiple comparisons test (normally distributed data). Individual data are depicted on top of boxplots showing median and interquartile range, as well as minimum and maximum values (indicated by error bars). Significance level for the analyses was set to α = 0.05. No significant differences were observed between the two groups. Significant differences between the respective time points and the pre-bedrest baseline are indicated. *HDL* high-density lipoprotein, *RVE* resistive vibration exercise.

The potential anti-atherogenic effects of HDL are well documented^6, 22^. We observed that three weeks of bedrest (day 21) significantly impaired HDL-cholesterol levels both in the bedrest only (*p* = 0.011), as well as in the bedrest plus resistive vibration exercise (RVE) (*p* = 0.005) groups (Fig. 1a, Table 1). Specifically, impaired HDL-cholesterol levels were already observed at one week of bedrest (day 7) (Fig. 1a). HDL-cholesterol levels did not recover at the first day post-bedrest (Fig. 1a). In addition to the profound changes in HDL-cholesterol, we observed that levels of the major HDL apolipoproteins, apoA-I (bedrest *p* = 0.001 and bedrest plus RVE *p* = 0.001 respectively) and apoA-II (bedrest *p* = 0.002 and bedrest plus RVE *p* = 0.001 respectively), as well as total plasma cholesterol (bedrest *p* = 0.001 and bedrest plus RVE *p* = 0.002 respectively), triglycerides (bedrest *p* = 0.014 and bedrest plus RVE *p* = 0.002 respectively) and low-density lipoprotein-cholesterol (bedrest *p* = 0.040 and bedrest plus RVE *p* = 0.029 respectively) were significantly decreased after three weeks of bedrest in both groups (Table 1). No change was observed in the content of HDL-associated serum amyloid A (SAA) (Table 1), a sensitive marker of inflammation^23^ and in the triglyceride/HDL-cholesterol ratio (see Supplementary Fig. S1 online).

**Table 1:** Clinical characteristics of study subjects.

	Pre-bedrest baseline	Day 21 of bedrest	*p*-value
**Bedrest group**			
N	11	11	
Total cholesterol (mg/dL)	143.5 ± 20.0	122.0 ± 21.2	0.001
HDL-cholesterol (mg/dL)	36.8 ± 6.7	30.0 ± 6.8	0.001
LDL-cholesterol (mg/dL)	90.2 ± 15.9	79.6 ± 16.1	0.040
Total triglyceride (mg/dL)	82.6 ± 19.8	62.2 ± 13.3	0.014
Plasma apoA-I (mg/dL)	120.4 ± 13.7	97.8 ± 14.9	0.001
Plasma apoA-II (mg/dL)	36.0 ± 4.2	30.4 ± 2.8	0.002
Plasma SAA (mg/dL)	0.5 (0.2–0.6)	0.3 (0.2–0.9)	0.624
**Bedrest plus RVE group**			
N	11	11	
Total cholesterol (mg/dL)	161.1 ± 28.1	130.2 ± 24.4	0.002
HDL-cholesterol (mg/dL)	38.3 ± 6.7	29.8 ± 5.0	0.001
LDL-cholesterol (mg/dL)	102.9 ± 22.2	87.4 ± 20.0	0.029
Total triglyceride (mg/dL)	99.8 ± 34.7	65.1 ± 16.8	0.002
Plasma apoA-I (mg/dL)	127.3 ± 16.6	97.3 ± 9.5	0.001
Plasma apoA-II (mg/dL)	39.1 ± 3.2	30.6 ± 3.1	0.001
Plasma SAA (mg/dL)	0.8 (0.5–1.0)	0.8 (0.5–1.0)	0.966

HDL-cholesterol, low-density lipoprotein cholesterol, total cholesterol, triglycerides, apoA-I, apoA-II and SAA were evaluated in plasma for the bedrest (n = 11) and the bedrest plus resistive vibration exersise (n = 11) groups. Data are presented as mean ± SD (normally distributed data), or as median with interquartile range (not normally distributed data). Differences between pre-bedrest baseline and day 21 of bedrest were analyzed either with the paired *t* test, two-tailed (for normally distributed data), or with the Wilcoxon matched-pairs signed rank test, two-tailed (for not normally distributed data). Significance level for the analyses was set to α = 0.05 and differences are indicated with the corresponding *p*-value.

*apoA-I* apolipoprotein A-I, *apoA-II* apolipoprotein A-II, *HDL* high-density lipoprotein, *LDL* low-density lipoprotein, *N* number of subjects, *RVE* resistive vibration exercise, *SAA* serum amyloid A.

### Effects of prolonged bedrest on HDL cholesterol efflux capacity

A key cardioprotective and anti-inflammatory property of HDL is their ability to remove cholesterol from macrophages^6^. We observed that three weeks of bedrest profoundly decreased HDL cholesterol efflux capacity (Fig. 1b). Resistive vibration exercise countermeasure during bedrest did not recover the cholesterol efflux capacity (Fig. 1b). A trend towards improved cholesterol efflux capacity was observed in the bedrest group during the first day post-bedrest (p = 0.079).

### Effects of prolonged bedrest on anti-oxidative capacity and HDL-associated enzyme activities

**Figure 2:**
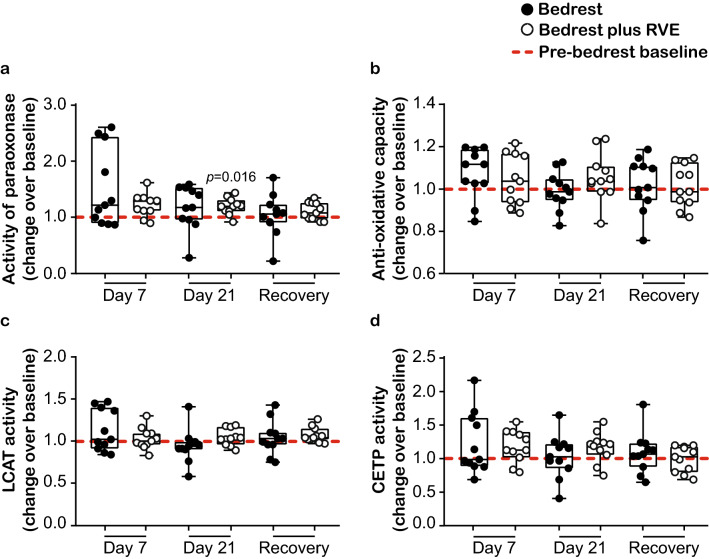
Effects of prolonged bedrest on anti-oxidative capacity and HDL-associated enzyme activities. HDL anti-oxidative capacity as well as activities of HDL-associated enzymes were evaluated for the bedrest (n = 11) and the bedrest plus RVE (n = 11) groups. (**a**) Arylesterase activity of paraoxonase was evaluated in apoB-depleted plasma using phenylacetate as substrate, in two independent experiments, measured in duplicates. (**b**) The anti-oxidative capacity of plasma was evaluated using the 2,2′-azinobis-(3-ethylbenzothiazoline-6-sulfonic acid) based assay, in two independent experiments, measured in duplicates. (**c**) Plasma LCAT and (**d**) CETP activities were evaluated with commercially available kits, in one experiment respectively, measured in duplicates. (**a**–**d**) Differences between pre-bedrest baseline and day 7 of bedrest, day 21 of bedrest and one day recovery, as well as differences between the two groups at each time point, were analyzed with RM one-way ANOVA using the Sidak’s multiple comparisons test (normally distributed data). Individual data are depicted on top of boxplots showing median and interquartile range, as well as minimum and maximum values (indicated by error bars). Significance level for the analyses was set to α = 0.05. No significant differences were observed between the two groups. Significant differences between the respective time points and the pre-bedrest baseline are indicated. *CETP* cholesterol ester transfer protein, *LCAT* lecithin-cholesterol acyltransferase, *RVE* resistive vibration exercise.

HDL-associated paraoxonase is an esterase with vascular protective properties^22^ and its activity has been related to cardiovascular risk^24^. We observed that the arylesterase activity of paraoxonase was not altered in the bedrest only group, while we found a trend towards increased activity in the bedrest plus RVE group (significant after three weeks, *p* = 0.016) (Fig. 2a). Bedrest did not alter the anti-oxidative capacity of plasma (Fig. 2b). Prompted by the profound effects of bedrest on HDL quantity and quality, we assessed LCAT and CETP activities, since both enzymes play a fundamental role in HDL metabolism^25^. Surprisingly, bedrest did not alter LCAT (Fig. 2c) and CETP (Fig. 2d) activities in either of the two groups studied.

### Functionality of HDL particles is not altered during bedrest

**Figure 3:**
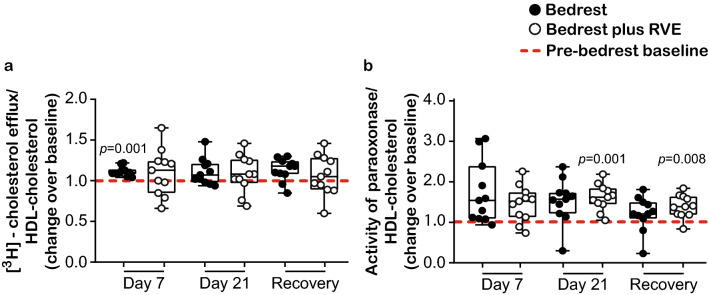
Functionality of HDL particles after adjustment for HDL-cholesterol. (**a**) The ability of HDL to promote [^3^H]-cholesterol efflux from macrophages and (**b**) the arylesterase activity of paraoxonase were adjusted for plasma HDL-cholesterol levels for the bedrest (n = 11) and the bedrest plus RVE (n = 11) groups. (**a**,**b**) Differences between pre-bedrest baseline and day 7 of bedrest, day 21 of bedrest and one day recovery, as well as differences between the two groups at each time point, were analyzed with RM one-way ANOVA using the Sidak’s multiple comparisons test (normally distributed data). Individual data are depicted on top of boxplots showing median and interquartile range, as well as minimum and maximum values (indicated by error bars). Significance level for the analyses was set to α = 0.05. No significant differences were observed between the two groups. Significant differences between the respective time points and the pre-bedrest baseline are indicated. *HDL* high-density lipoprotein, *RVE* resistive vibration exercise.

The cholesterol efflux capacity is an integrative measure of both HDL quantity and quality. A significant association was observed between HDL cholesterol efflux capacity and HDL-cholesterol (Pearson’s *r* = 0.473, *p* = 0.001, see Supplementary Fig. S2a online). To test whether the functionality of HDL particles (besides HDL quantity) is impaired during bedrest, we adjusted cholesterol efflux capacity for HDL-cholesterol values of the respective subjects. It is noteworthy that after the adjustment, the observed effects of bedrest on HDL cholesterol efflux capacity were abolished, indicating that the reduction of HDL-cholesterol levels during bedrest leads to an impairment of cholesterol efflux capacity (Fig. 3a). Interestingly, even a slight increase in the adjusted cholesterol efflux capacity was observed during bedrest (significant on day 7 in the bedrest only group, *p* = 0.001) (Fig. 3a). As expected, HDL cholesterol efflux capacity was also associated with apoA-I levels (Pearson’s *r* = 0.864, *p* = 0.001) and adjustment of cholesterol efflux for apoA-I abolished the observed effects (see Supplementary Fig. S2b online).

In the bloodstream, paraoxonase is bound to HDL, which not only stabilizes the enzyme, but also provides the hydrophobic environment necessary for its function^26^. Previous studies have reported mixed results, with some studies associating lower HDL-cholesterol levels with decreased paraoxonase activity^26^, while others reporting that paraoxonase activity is negatively correlated with HDL-cholesterol levels^27^. To investigate whether the paraoxonase activity per HDL particle is altered during bedrest, we adjusted the activity of paraoxonase against HDL-cholesterol values of the respective subjects. Interestingly after the adjustment for HDL-cholesterol, the observed effects of bedrest on paraoxonase activity appeared to be increased (day 21 and one day recovery in the bedrest plus RVE group, *p* = 0.001 and *p* = 0.008, respectively) suggesting that bedrest does not impair HDL quality, but affects HDL quantity (Fig. 3b). We observed only a trend of an association between paraoxonase activity and apoA-I levels (p = 0.090).

## Discussion

To our knowledge, this is the first study evaluating multiple metrics of HDL composition and function in healthy male volunteers partricipating in a bedrest study. We observed that bedrest leads to impaired HDL cholesterol efflux capacity along with decreased levels of apoA-I, apoA-II, HDL-cholesterol, low-density lipoprotein-cholesterol and triglycerides. Our results are of importance, given that HDL cholesterol efflux capacity predicts coronary heart disease regardless of age and gender^6, 28^, suggesting that a reduced cholesterol efflux capacity during long bedrest may increase cardiovascular risk in older subjects.

Importantly, lipoproteins are not only lipid transporters but also display critical functions in many aspects of immunity, for example HDL have a high affinity for binding and neutralizing pathogen-associated lipids^8, 9^. Therefore, reduced HDL-cholesterol levels in bedridden patients may increase risk for infections even at short-term. Cholesterol efflux capacity is markedly impaired in septic patients^29, 30^ and physical inactivity, inadequate exercise and an overall sedentary lifestyle are associated with impaired immune response^31^ and increased risk of viral infections^31^ and sepsis^32, 33^. Low levels of HDL-cholesterol and low-density lipoprotein-cholesterol are associated with poor clinical outcomes and increased risk of infectious disease and survival, respectively^34–36^, while in critically ill and especially in septic patients, a reduction in lipid and lipoprotein levels has been well documented^37^, in particular apoA-I, apoA-II^15, 30^, HDL and low-density lipoprotein^30, 38, 39^. Prolonged bedrest increases the risk of hospital-acquired pneumonia, which is often caused by bacterial infection^3^. In particular, a negative correlation between HDL concentration^40^ or apoA-I^41^ and mortality in human sepsis has been described, while some authors suggest HDL to be an early marker of sepsis severity^12, 40^.

Interestingly, we observed no effect of bedrest on anti-oxidative capacity of plasma and arylesterase activity of paraoxonase. When adjusting cholesterol efflux capacity and paraoxonase activity of apoB-depleted plasma against HDL-cholesterol levels, we observed that bedrest apparently did not impair the quality of individual HDL particles. Bedrest appears to only affect HDL quantity, which was linked to a lower cholesterol efflux capacity of apoB depleted plasma.

Our results are in line with a previous bedrest study, including healthy male volunteers performing resistive vibration exercise along with amino acid supplementation, showing that HDL-cholesterol and total cholesterol were decreased during bedrest^19^. Other head-down bedrest studies reported mixed results. Specifically, in a 60 days’ head-down bedrest study involving healthy female volunteers, bedrest appeared to increase the apoB/apoA-I ratio^42^, while other head-down bedrest studies reported increased levels of triglycerides^20, 43^, but no change in low-density lipoprotein-cholesterol^43^. In contrast, we observed markedly decreased levels of low-density lipoprotein-cholesterol and triglycerides after three weeks of bedrest in both groups studied. In a previous 20 days’ bedrest study involving healthy young male and female volunteers, bedrest appeared to enhance LCAT activity at day 11 and 21 of the study, while no change was observed in CETP activity^20^. Moreover, in a 35 days’ head-down bedrest study involving healthy young male volunteers, plasma CETP concentrations were increased during bedrest^43^. In the present study, we observed that bedrest did not alter LCAT and CETP activities in either of the two groups studied. Variations in duration and design of the bedrest studies may explain the mixed results.

Some limitations have to be noted. The guidelines for standardization of bedrest studies are very strict and potential participants need not only to fulfil a number of criteria, but also to undergo initial screenings prior to inclusion in the bedrest study; therefore bedrest studies include a limited number of participants. Although our results are robust, the limited number of participants in this study could affect the statistical power of the analyses. Finally, while the pre-bedrest baseline values were similar between the two groups in this crossover study, we cannot exclude a carry-over effect.

In conclusion, we observed that prolonged bedrest markedly affects HDL quantity associated with decreased cholesterol efflux capacity. Resistive vibration exercise countermeasure did not correct the HDL cholesterol efflux capacity. Intensive exercise has been shown to increase levels of HDL-cholesterol^44, 45^, so it would be of importance to examine, whether a more frequent exercise schedule during bedrest increases HDL-cholesterol levels and functionality. Our results support the view that unnecessary and/or prolonged bedrest should be avoided. Further studies are needed to confirm our results.

## Materials and methods

### Materials

J774.2 BALB/C monocyte macrophage cell line (85011428), Polyethylene Glycol solution (P1458), Sandoz 58-035 (S9318), 8-(4-Chlorophenylthio)adenosine 3́, 5́-cyclic monophosphate sodium salt (C3912), Bovine Serum Albumin (A8806), Phenyl Acetate 99% (108723), Calciumchlorid-Dihydrat (102382), 2,2́-Azino-bis(3-ethylbenzothiazoline-6-sulfonic acid) diammonium salt (A1888), ( ±)-6-Hydroxy-2,5,7,8-tetramethylchromane-2-carboxylic acid (238813), Potassium Persulfate (216224), LCAT activity assay kit (MAK107) and CETP activity assay kit (MAK106) were obtained by Merck, Darmstadt, Germany. Glycin (3908.2) and TRIS (AE15.2) were obtained by Carl-Roth, Karlsruhe, Germany. Cell Culture Multiwell Plate 48 Well PS Clear Cellstar TC (677180) was obtained from Greiner BIO-ONE, Kremsmünster, Austria. Dulbecco’s Modified Eagle’s Medium DMEM high glucose (41965039) and Fetal Bovine Serum FBS qualified Brazil (10270106) were obtained from Thermo Fisher Scientific, Waltham, Massachusetts, USA. Penicillin–Streptomycin 10,000 U/ml Penicillin 10 mg/ml Streptomycin (P06-07100) was obtained from PAN-Biotech, Aidenbach, Germany. Cholesterol [1,2-3H(N)] (ART0255) was obtained from ARC, St. Louis, MO, USA. Apolipoprotein A-I (171029910021), Total Cholesterol (113009910917), Triglycerides (157109910917) and High-Density Lipoprotein (HDL)-cholesterol (135219910930) were obtained from DiaSys Diagnostic Systems GmbH, Holzheim, Germany. Apolipoprotein A-II (KAI-003) was obtained from Kamiya Biomedical Company, Seattle, WA, USA. SAA Human ELISA kit (EHSAA1) was obtained from Invitrogen (Carlsbad, California, USA).

### Ethical approvals

The ‘Medium duration Nutrition and vibration eXercise’ bedrest study, with and without countermeasures, took place at the Institute for Space Medicine and Physiology, MEDES Clinique d’Investigation, Toulouse, France, as described previously^21^. The study was conducted under the leadership of the French and European Space Agencies. Ethical approval was obtained from the local ethics committee at MEDES, Toulouse, France. This study was carried out in accordance with the Declaration of Helsinki guidelines for research on human subjects (1989). All subjects provided written informed consent prior to participation in this study. The written consent forms are stored at the MEDES clinic in Toulouse.

### Study subjects and blood sampling

The current investigation is based on the re-analysis of the plasma available from the donors involved in the ‘Medium duration Nutrition and vibration eXercise’ bedrest study, as previously reported^21, 46^. Eleven medically and psychologically healthy male volunteers (age 34.3 ± 8.3 years and BMI 22.4 ± 1.7, mean ± SD) were recruited to undergo 21 days of 6 degrees head-down bedrest. Subject characteristics, medical check-up, inclusion and exclusion criteria, as well as dropout criteria and study design have been described previously^21^. In brief, blood was drawn, to isolate plasma, 5 days before commencement of bedrest (pre-bedrest baseline), on day 7 and 21 of bedrest and at the first day post-bedrest (recovery). Plasma was isolated, as described^46^. The volunteers were subdivided into the bedrest only group, the bedrest plus resistive vibration exercise group and the bedrest plus resistive vibration exercise plus high-protein/high-calorie diet group randomly, and each volunteer carried out the three protocols (having a 4 month interval between each bedrest session to recuperate)^21^. In our study, samples from the bedrest only and the bedrest plus resistive vibration exercise groups were analysed.

### Bedrest plus resistive vibration exercise countermeasure

During the bedrest period, the eleven volunteers of the bedrest plus resistive vibration exercise group followed a controlled standard diet and performed physical training twice per week^21^. Following this fixed schedule, they were transferred, while on the 6 degrees head-down position, onto a vibrating platform exercising leg muscles while absorbing the up-and-down motion on a machine specially designed for this purpose. The volunteers were pulled onto the plates with a force equivalent to 100–200 kg, while performing upside-down leg-presses for a few minutes^21^.

### Bedrest group (sedentary; only bedrested)

The eleven volunteers of the bedrest group followed a controlled standard diet without physical training^21^.

### Preparation of apo-B-depleted plasma

ApoB-depleted plasma was prepared by addition of polyethylenglycol (40 μL, 20% in 200 mmol/L Glycine buffer) to plasma (100 μL) with gentle mixing. Consequently, plasma was incubated at room temperature for 20 min, followed by centrifugation (10,000 rpm, 20 min, 4 °C), after which the supernatant was recovered^47^. Samples were stored at -70° C until use.

### Biochemical quantification of plasma proteins and lipids

Plasma apoA-I and apoA-II were determined by immunoturbidimetry and SAA was determined by enzyme-linked immunosorbent assay, as described^48^. All lipoprotein analyses were performed on an Olympus AU640 analyzer (Beckman Coulter, Brea, CA). Total cholesterol, triglycerides and HDL-cholesterol were determined by commercially available kits, as described^48^. Low-density lipoprotein cholesterol was calculated according to the Friedewald equation using HDL-cholesterol values measured in plasma.

### HDL cholesterol efflux capacity

HDL cholesterol efflux capacity of apoB-depleted plasma was determined using 8-(4-chlorophenylthio)-cyclic adenosine monophosphate stimulated J774.2 cells, as described^6^. Briefly, J774.2 macrophages were maintained in Dulbecco’s Modified Eagle’s Medium in the presence of fetal bovine serum (10%) and penicillin/streptomycin (1%). Cells were plated on 48-well plates and labeled for 24 h with [^3^H]-cholesterol (0.5 μCi/mL) in Dulbecco’s Modified Eagle’s Medium supplemented with fetal bovine serum (2%) and penicillin/streptomycin (1%). Labeled macrophages were incubated in the presence of 8-(4-chlorophenylthio)-cyclic adenosine monophosphate (0.3 mmol/L) to stimulate ATP-binding cassette transporter A1 expression. After labeling, cells were rinsed and equilibrated in serum free Dulbecco’s Modified Eagle’s Medium containing bovine serum albumin (2 mg/mL) for 2 h.[^3^H]-cholesterol efflux was determined by incubating cells for 3 h with serum free Dulbecco’s Modified Eagle’s Medium containing apoB-depleted plasma (2.8%). HDL cholesterol efflux capacity is expressed as radioactivity in the cell culture supernatant relative to total radioactivity (in the cell culture supernatant and cells) of two independent experiments, measured in duplicates. All steps were performed in the presence of acyl coenzyme A cholesterol acyltransferase inhibitor Sandoz 58–035 (2 μg/mL).

### Arylesterase activity of paraoxonase

Arylesterase activity of paraoxonase was determined in apoB-depleted plasma by a photometric assay using phenylacetate as substrate, as described^48^ with modifications. Specifically, apoB-depleted plasma (3 μL of 1:10 dilution) was added to 200 μL buffer containing Tris (100 mmol/L), CaCl_2_ (pH 8.0) (2 mmol/L) and phenylacetate (1 mmol/L). The rate of hydrolysis of phenylacetate was monitored by the increase of absorbance at 270 nm and readings were taken every 30 s at room temperature to generate a kinetic plot. The slope from the kinetic chart was used to determine ΔAb270nm/min. Enzymatic activity was calculated with the Beer-Lambert Law from the molar extinction coefficient of 1,310 L × mol − 1 × cm − 1 for phenylacetate. Activities were calculated from the slopes of the kinetic chart of two independent experiments, measured in duplicates.

### Anti-oxidative capacity of plasma

The anti-oxidative capacity of plasma was assessed using the 2,2´-azinobis-(3-ethylbenzothiazoline-6-sulfonic acid) based assay, as described^49^, in two independent experiments, measured in duplicates.

### LCAT and CETP activities

LCAT and CETP activities of plasma were measured in duplicates, in one independent experiment respectively, using commercially available kits, as previously described^48^.

### Statistical analysis

Data were normalized to the corresponding pre-bedrest baseline values and tested for normality using the Shapiro-Wilk normality test^50^. Differences between pre-bedrest baseline and day 21 of bedrest were analyzed either with the paired *t* test, two-tailed (normally distributed data)^51^, or with the Wilcoxon matched-pairs signed rank test, two-tailed (not normally distributed data)^52^. Differences between pre-bedrest baseline and day 7 of bedrest, day 21 of bedrest and one day recovery, as well as differences between the two groups at each time point, were analyzed with RM one-way ANOVA using the Sidak’s multiple comparisons test (normally distributed data)^53^. Significance level for the analyses was set to α = 0.05 and significant differences are indicated with the corresponding *p*-value. Individual data are depicted on top of boxplots showing median and interquartile range, as well as minimum and maximum values (indicated by error bars). Correlation between HDL-cholesterol levels and HDL cholesterol efflux capacity was determined using the Pearson’s correlation coefficient *r* (normally distributed data) and is depicted in scatter plot. Statistical analyses were performed using GraphPad Prism (Version 8.0.1; GraphPad Software) and SPSS Statistics (Version 23). Graph design was performed using GraphPad Prism (Version 8.0.1; GraphPad Software; https://www.graphpad.com) and image processing using Adobe Illustrator CC 2020 (Version 24.0.2; https://www.adobe.com/products/illustrator.html).

## Supplementary information

Supplementary information

## Data Availability

The datasets generated during and/or analysed during the current study are available from the corresponding author on reasonable request.

## Abbreviations

Apo: Apolipoprotein
CETP: Cholesteryl ester transfer protein
HDL: High-density lipoprotein
LCAT: Lecithin-cholesterol acyltransferase
LDL: Low-density lipoprotein
RVE: Resistive vibration exercise
SAA: Serum amyloid A
