# Supplementary material for: Prolonged bedrest reduces plasma high-density lipoprotein levels linked to markedly suppressed cholesterol efflux capacity
Source: Sci Rep. 2020 Sep 14;10:15001. doi: 10.1038/s41598-020-71921-y (PMC7490699; doi:10.1038/s41598-020-71921-y)
Supplement: Supplementary file 1 — Supplementary information [file 41598_2020_71921_MOESM1_ESM.pdf]

Supplementary Information

Prolonged bedrest reduces plasma high-density lipoprotein levels linked to markedly suppressed cholesterol efflux capacity

Athina Trakaki [1], Hubert Scharnagl [2], Markus Trieb [1, 3], Michael Holzer [1], Helmut Hinghofer-Szalkay [4], Nandu Goswami [4*] and Gunther Marsche [1, 3*]

Affiliations:
[1] Division of Pharmacology, Otto Loewi Research Center for Vascular Biology, Immunology and Inflammation, Medical University of Graz, Universitätsplatz 4, 8010 Graz, Austria.
[2] Institute of Medical and Chemical Laboratory Diagnostics, Medical University of Graz, Auenbruggerplatz 15, 8036 Graz, Austria.
[3] BioTechMed Graz, Mozartgasse 12/II, 8010 Graz, Austria.
[4] Division of Physiology, Otto Loewi Research Center for Vascular Biology, Immunology and Inflammation, Neue Stiftingtalstrasse 6/D-5, Medical University of Graz, Graz, Austria.

* *Correspondence*:
Gunther Marsche; Division of Pharmacology, Otto Loewi Research Center for Vascular Biology, Immunology and Inflammation, Medical University of Graz, Universitätsplatz 4, 8010 Graz, Austria; phone: + 43 316 385 74128
email: gunther.marsche@medunigraz.at

Nandu Goswami; Division of Physiology, Otto Loewi Research Center for Vascular Biology, Immunology and Inflammation, Medical University of Graz, Neue Stiftingtalstraße 6/D05, 8010, Graz, Austria; phone: +43 316 385 73852
email: nandu.goswami@medunigraz.at

Supplementary Figures

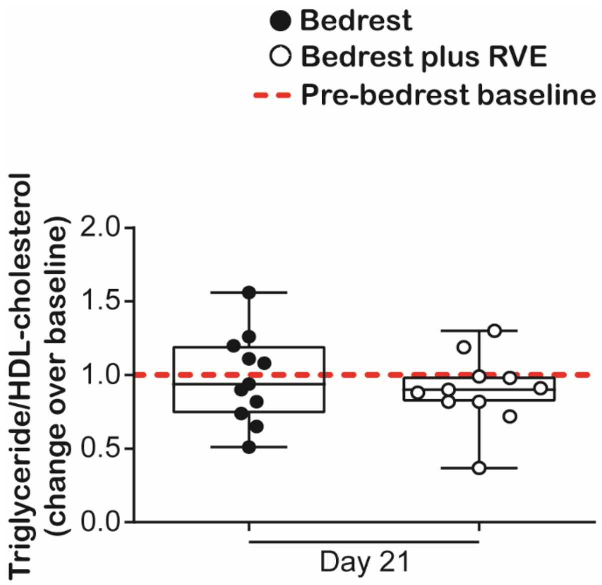

Supplementary Figure S1. Effects of prolonged bedrest on triglyceride/HDL-cholesterol ratio

The triglyceride/HDL-cholesterol ratio was evaluated for the bedrest (n = 11) and the bedrest plus RVE (n = 11) groups at pre-bedrest baseline and day 21 of bedrest. Differences between pre-bedrest baseline and day 21 of bedrest, as well as differences between the two groups at day 21, were analyzed with RM one-way ANOVA using the Sidak's multiple comparisons test (normally distributed data). Individual data are depicted on top of boxplots showing median and interquartile range, as well as minimum and maximum values (indicated by error bars). Significance level for the analyses was set to α = 0.05. No significant differences were observed between the two groups or between pre-bedrest baseline and day 21 for each group. Abbreviations represent: HDL, high-density lipoprotein; RVE, resistive vibration exercise.

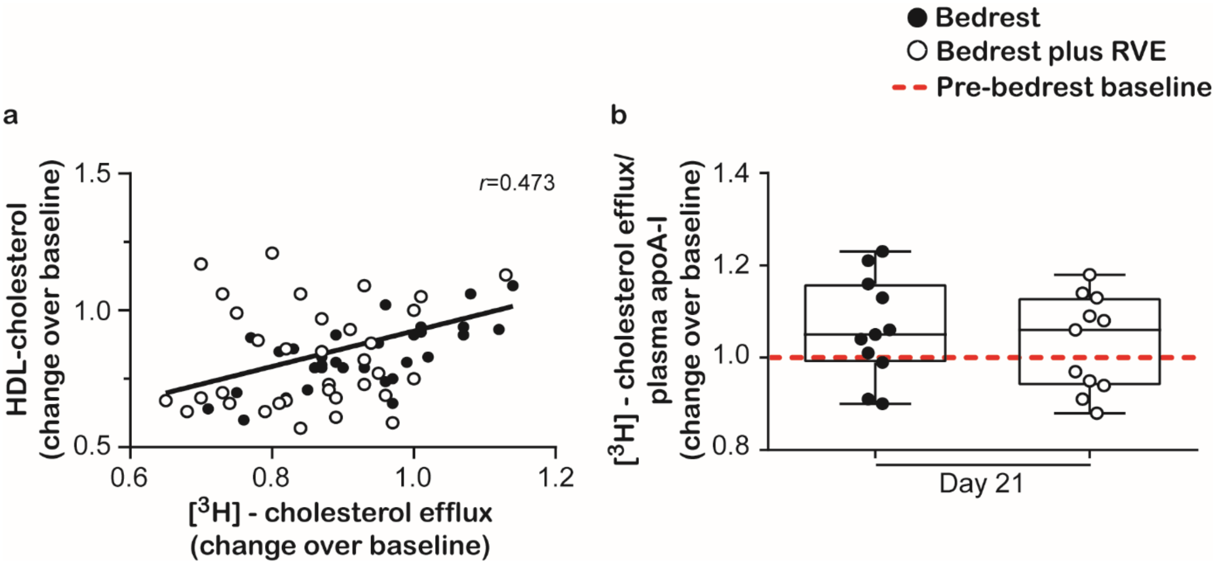

Supplementary Figure S2. Correlation of HDL-cholesterol with HDL cholesterol efflux capacity and functionality of HDL particles after adjustment for plasma apoA-I

(a) HDL-cholesterol levels for the bedrest (n = 11) and the bedrest plus RVE (n = 11) groups were associated with HDL cholesterol efflux capacity. Data are indicated in scatter plot and the Pearson correlation coefficient *r* is noted (normally distributed data). Significance level for the analyses was set to $\alpha = 0.05$. (b) The ability of HDL to promote [^{3}H]-cholesterol efflux from macrophages was adjusted for plasma apoA-I levels for the bedrest (n = 11) and the bedrest plus RVE (n = 11) groups at pre-bedrest baseline and day 21 of bedrest. Differences between pre-bedrest baseline and day 21 of bedrest, as well as differences between the two groups at day 21, were analyzed with RM one-way ANOVA using the Sidak's multiple comparisons test (normally distributed data). Individual data are depicted on top of boxplots showing median and interquartile range, as well as minimum and maximum values (indicated by error bars). Significance level for the analyses was set to $\alpha = 0.05$. No significant differences were observed between the two groups or between pre-bedrest baseline and day 21 for each group. Abbreviations represent: apo, apolipoprotein; HDL, high-density lipoprotein; RVE, resistive vibration exercise.
